# Gene expression comparison of resistant and susceptible Atlantic salmon fry challenged with Infectious Pancreatic Necrosis virus reveals a marked contrast in immune response

**DOI:** 10.1186/s12864-016-2600-y

**Published:** 2016-04-11

**Authors:** Diego Robledo, John B. Taggart, Jacqueline H. Ireland, Brendan J. McAndrew, William G. Starkey, Chris S. Haley, Alastair Hamilton, Derrick R. Guy, Jose C. Mota-Velasco, Almas A. Gheyas, Alan E. Tinch, David W. Verner-Jeffreys, Richard K. Paley, Georgina S. E. Rimmer, Ian J. Tew, Stephen C. Bishop, James E. Bron, Ross D. Houston

**Affiliations:** The Roslin Institute and Royal (Dick) School of Veterinary Studies, University of Edinburgh, Midlothian, EH25 9RG UK; Departamento de Genética, Facultad de Biología, Universidad de Santiago de Compostela, Santiago de Compostela, 15782 Spain; Institute of Aquaculture, School of Natural Sciences, University of Stirling, Stirling, FK9 4LA UK; Landcatch Natural Selection Ltd., 15 Beta Centre, Stirling University Innovation Park, Stirling, FK9 4NF UK; Centre for Environment, Fisheries and Aquaculture Science (Cefas), Weymouth, DT4 8UB UK

**Keywords:** *Salmo salar*, virus, transcriptome, infectious pancreatic necrosis, disease resistance, interferon, innate immunity, aquaculture

## Abstract

**Background:**

Infectious Pancreatic Necrosis (IPN) is a highly contagious birnavirus disease of farmed salmonid fish, which often causes high levels of morbidity and mortality. A large host genetic component to resistance has been previously described for Atlantic salmon (*Salmo salar* L.), which mediates high mortality rates in some families and zero mortality in others. However, the molecular and immunological basis for this resistance is not yet fully known. This manuscript describes a global comparison of the gene expression profiles of resistant and susceptible Atlantic salmon fry following challenge with the IPN virus.

**Results:**

Salmon fry from two IPNV-resistant and two IPNV-susceptible full sibling families were challenged with the virus and sampled at 1 day, 7 days and 20 days post-challenge. Significant viral titre was observed in both resistant and susceptible fish at all timepoints, although generally at higher levels in susceptible fish. Gene expression profiles combined with gene ontology and pathway analyses demonstrated that while a clear immune response was observed in both resistant and susceptible fish, there were striking differences between the two phenotypes. The susceptible fish showed marked up-regulation of genes related to cytokine activity and inflammatory response that evidently failed to protect against the virus. In contrast, the resistant fish demonstrated a less pronounced immune response including up-regulation of genes relating to the M2 macrophage system.

**Conclusions:**

While only the susceptible phenotype shows appreciable mortality levels, both resistant and susceptible fish can become infected with IPNV. Susceptible fish are characterized by a much larger, yet ineffective, immune response, largely related to cytokine and inflammatory systems. Resistant fish demonstrate a more moderate, putative macrophage-mediated inflammatory response, which may contribute to their survival.

**Electronic supplementary material:**

The online version of this article (doi:10.1186/s12864-016-2600-y) contains supplementary material, which is available to authorized users.

## Background

Infectious pancreatic necrosis virus (IPNV) is a pathogen of salmonid fish which can cause high mortality and morbidity of cultured Atlantic salmon (*Salmo salar* L.) and rainbow trout (*Oncorhynchus mykiss*) and is responsible for serious economic losses to the aquaculture industry. IPNV forms part of the genus *Aquabirnavirus* and is a member of the Birnaviridae family, characterized by a bi-segmented double-stranded RNA genome. The clinical symptoms of IPNV infection include a swollen abdomen or eyes, darkening of the skin, pancreas necrosis and spiral swimming and the disease may eventually result in the death of infected hosts. In Atlantic salmon, outbreaks of the disease typically occur in two distinct windows of the production cycle; as newly-hatched fry at first feeding and in post-smolts during the months following transfer to seawater [1]. Vaccination can be used to protect post-smolt fish [2], but the control of freshwater outbreaks is dependent upon biosecurity in hatcheries and the level of innate resistance of the salmon fry. In this freshwater fry phase of the salmon life cycle, IPN outbreaks can result in near-complete population losses [1].

There is a large and significant host genetic component to variation in IPN mortality levels at both stages of the salmon lifecycle [3–5]. In addition, a quantitative trait locus (QTL) was demonstrated to have a major effect on IPN mortality in the seawater environment [6], and this QTL was subsequently confirmed in freshwater and seawater in both Scottish [7–9] and Norwegian [10, 11] populations. This major QTL results in a marked difference in mortality level (up to 100 %) between homozygous susceptible and homozygous resistant fish within and across families, with evidence for partial dominance of the resistance allele [8, 11]. As a result of the substantial genetic variation in host resistance, selective breeding for IPNV resistance has been effective in commercial aquaculture populations through both family and marker-based selection [5, 8, 10, 11]. Recently, Moen et al. [11] discovered SNPs associated with the putative QTL genotype (r^2^ 0.57 – 0.58) in the cadherin-1 gene (CDH1) gene which encodes a protein that co-locates with the IPN virus in liver cells and can bind to the IPN virus in vitro. These results suggest a possible role for CDH1 in the entry of the virus to host cells and that a non-synonymous SNP in the CDH1 gene may form part of the underlying mechanism of the QTL.

The host response to IPNV infection has been studied in salmonid fish and associated cell lines, and markers of type I and type II interferon responses are typically observed [12–15]. Further, Skjesol et al. [16] studied the host response to IPNV isolates of high and low virulence and demonstrated that both mortality levels and expression of key host immune response genes were positively associated with viral replication. Recent studies have also examined the differential gene expression response to infection between (partially) resistant and susceptible fish. For example, Cofre et al. [17] demonstrated that the expression of several pro-inflammatory genes and transcription factors was significantly higher in the head kidney of resistant fish. Most recently, Reyes-López et al. [18] studied head kidney gene expression profiles of resistant and susceptible salmon fry full-sibling families and suggested that a limited and prolonged immune response is associated with resistance while an acute short response is characteristic of susceptible fish.

In the current study, a series of IPNV challenges and microarray interrogations was undertaken to examine and contrast the transcriptome profile of IPNV-challenged whole fry from two IPN-susceptible families and two IPN-resistant families at 1 day, 7 days and 20 days post-challenge. Family- and timepoint-matched mock-challenged control fish were used as a baseline for comparison. An understanding of the differences in host response between resistant and susceptible genotypes is critical to advancing our understanding of the functional basis of genetic resistance to IPNV in salmon, and providing a more general perspective on the question of how host resistance can act to ameliorate viral pathogenesis.

## Results

### Disease challenge experiments

**Fig. 1:**
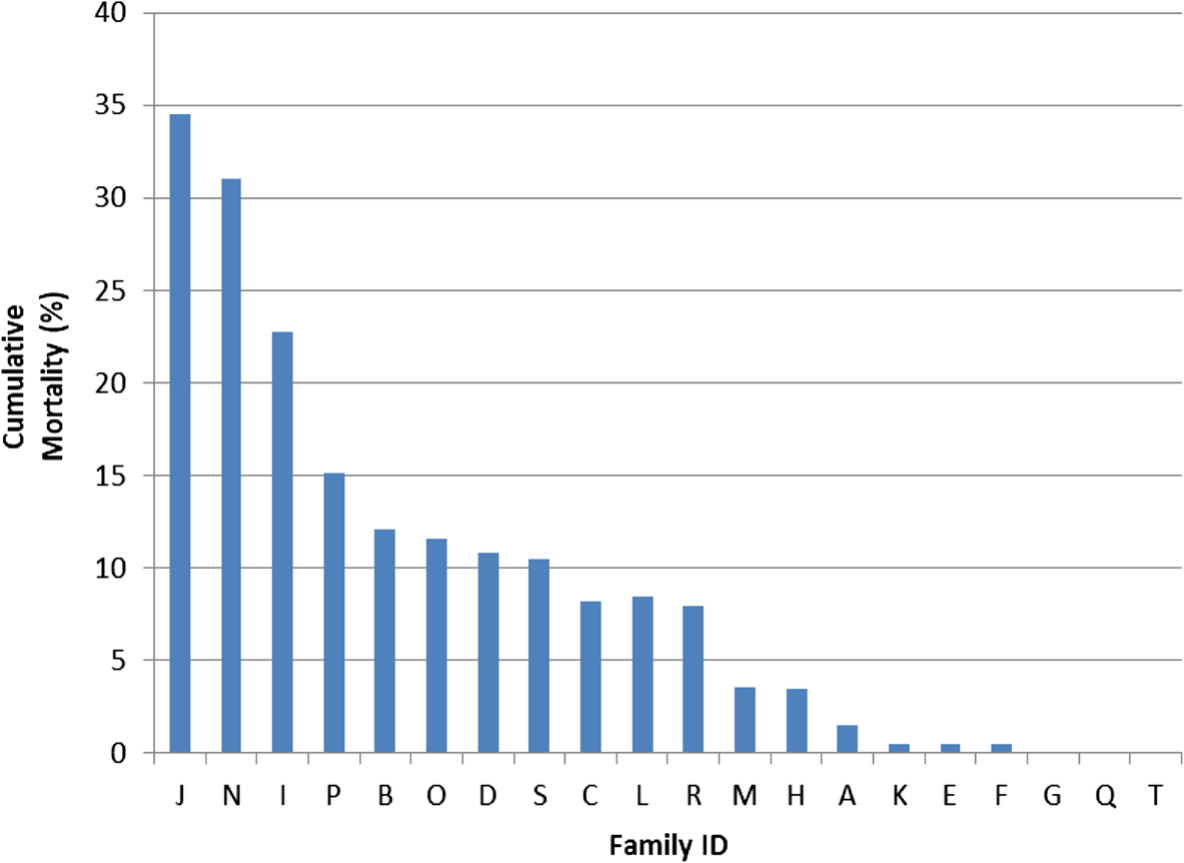
Family cumulative mortality. Cumulative mortality for the twenty families challenged with IPNV in the experiment described in [8]. The values are the mean of two replicate tanks. For the purposes of the subsequent gene expression challenge experiments on full siblings of these fish, families J and N were designated susceptible, and families Q and T resistant

To evaluate the difference in gene expression profiles between resistant and susceptible salmon fry, a subset of the most susceptible (*n* = 2) and the most resistant families (*n* = 2) from a previous study were examined. These families were selected based on an earlier IPNV immersion challenge experiment described by Houston et al. [8]. The family-specific mortality levels (averaged across the two replicate challenge tanks) are shown in Fig. 1. Families J and N were chosen as the ‘genetically susceptible’ families (mean mortality 33 %) and families Q and T were chosen as the ‘genetically resistant’ families (mean mortality 0 %). IPNV immersion challenges for the gene expression study commenced immediately after these initial challenge experiments, using full siblings of the fry used in the mortality study. Full details of the challenge protocol are given below in ‘Methods’.

### Family comparison

**Fig. 2:**
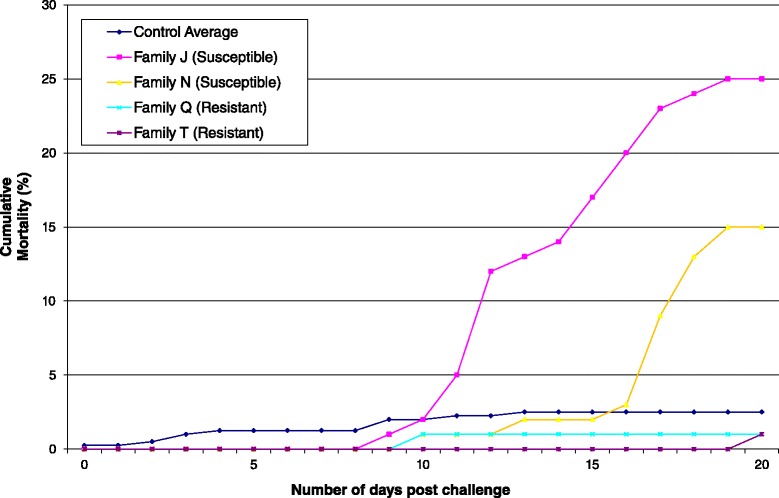
Mortalities for the four selected families. Cumulative mortality levels (average across two replicate tanks) in the four families from the tanks terminated at 20 days post-challenge in the current study. The control value is averaged across all families

For each family, six replicate tanks were challenged with IPNV alongside six control, mock-challenged tanks (two challenge tanks and two control tanks per timepoint, *n* = 50 per tank). At 1 day, 7 days and 20 days post-IPNV-challenge, the tanks were terminated and all surviving fry were sampled for RNA extraction and subsequent transcriptomics. The mortality profiles of the families matched expectations based on the earlier challenge experiment on their full siblings; i.e., families J and N showed significant mortality while families Q and T did not. As expected, there was also negligible mortality in the mock challenged tanks (Fig. 2). Note that these experiments were stopped at 20 days post infection; hence susceptible families do not reach the previously observed mortalities over 30 %, but the expected difference in mortality profile between the resistant and susceptible families is still observed (Fig. 2).

### IPNV virology

**Fig. 3:**
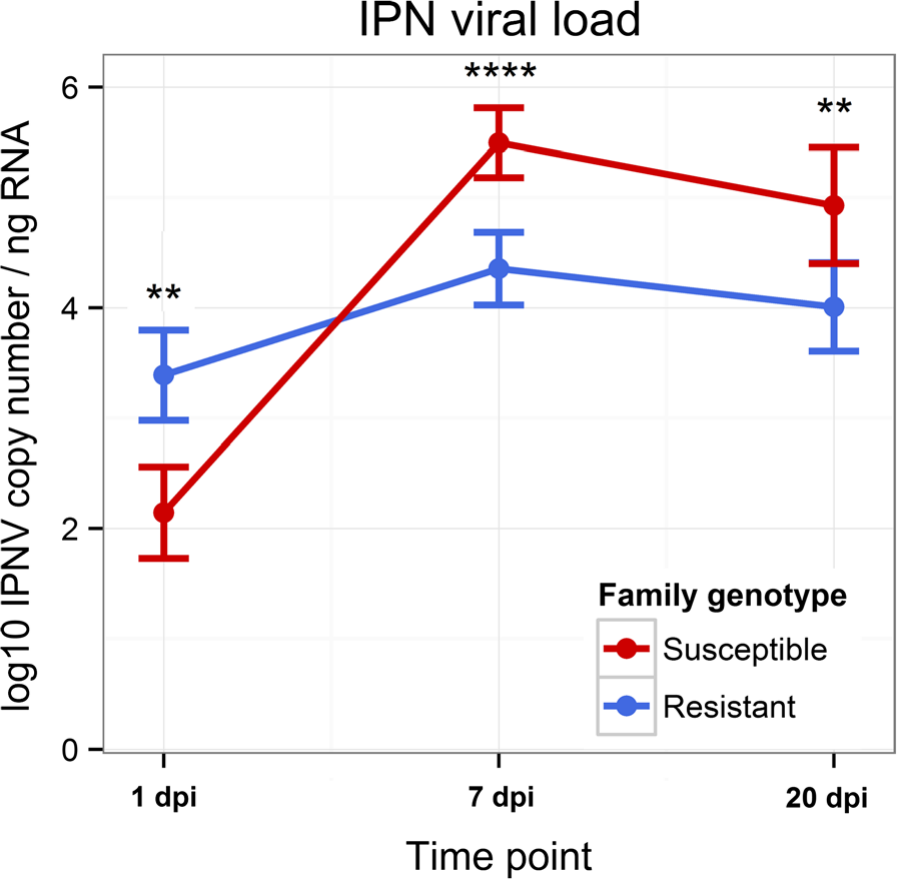
IPNV viral load in resistant and susceptible families. Mean and standard error log10 IPNV copy number per ng total RNA for resistant and susceptible samples at 1, 7 and 20 days post infection. Significance symbols correspond to the p-values for Mann–Whitney *U* test between resistant and susceptible samples (* p ≤ 0.05; ** p ≤ 0.01; *** p ≤ 0.001; **** p ≤ 0.0001)

Real-time PCR results revealed the presence of IPNV in both challenged resistant and susceptible families at all timepoints post infection (Fig. 3). Viral load was higher on average in susceptible fish than resistant fish at 7 and 20 days post infection (dpi) (10^4^ vs 10^5^ IPNV particles per ng of total RNA). Viral load was lower at 20 dpi than 7 dpi for susceptible fish, which might be explained by the mortality observed in these families from around 10 dpi, which is likely to have resulted in removal of fish with the highest viral load. These results demonstrate that animals with disparate genetic resistance can become infected and that viral infection and replication occurs in resistant genotypes, implying that genetic resistance cannot be entirely due to an inability of the virus to enter the cells of the host.

### Microarray profiling of gene expression

**Fig. 4:**
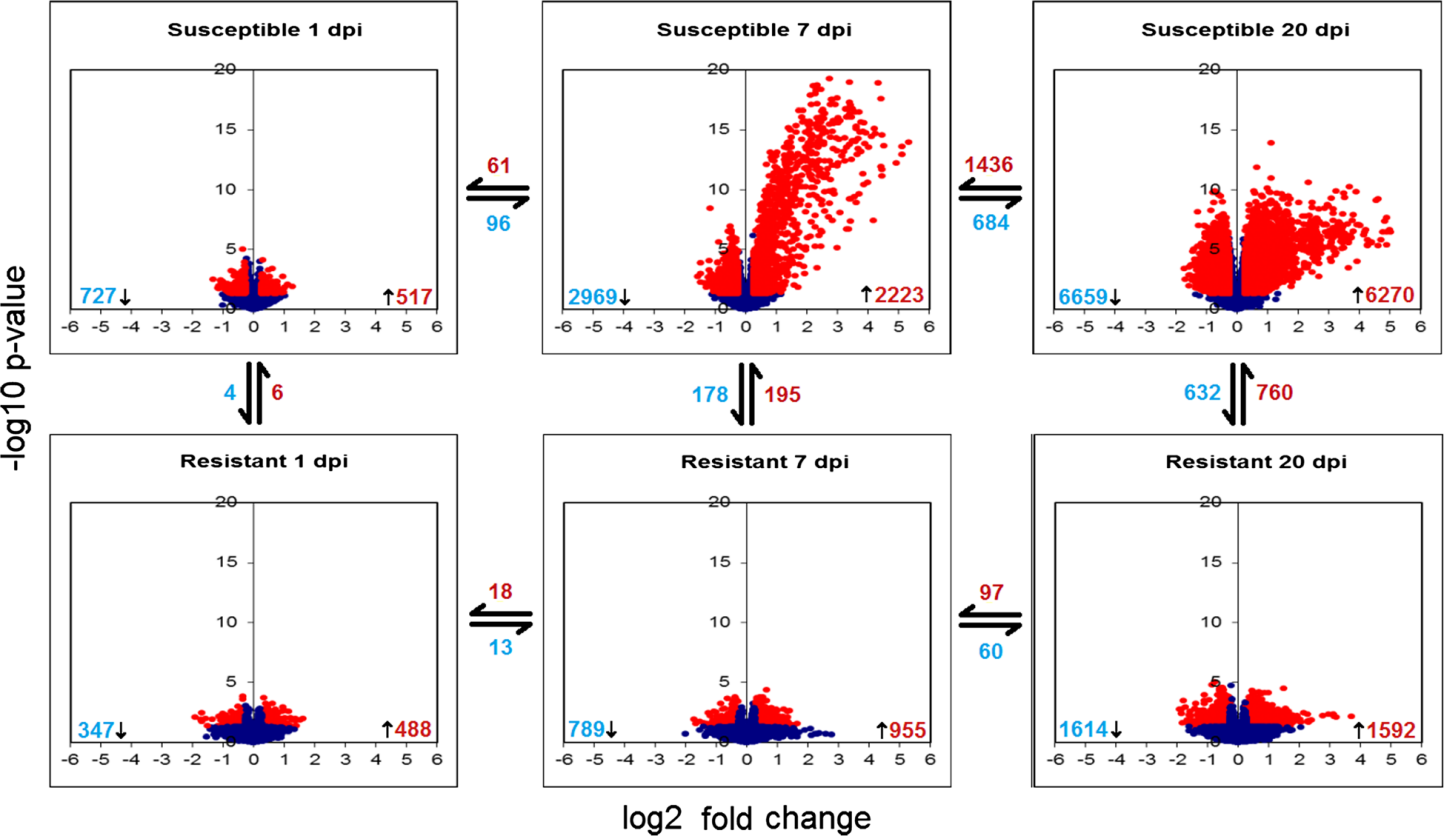
Volcano plots of global gene expression response in resistant and susceptible families following IPNV challenge. Volcano plots of the log2 fold change vs. log10 p-value of every transcript for each of the six comparisons are shown. Transcripts with p values < 0.05 (significant) are shown in red, while those with p values > 0.05 are shown in blue. The number of significant down-regulated (blue) and up-regulated (red) transcripts for each comparison are shown in the corners of each volcano plot. Likewise, the number of common down-regulated and up-regulated genes between each comparison are shown in the space between their volcano plots

A broad-level gene expression comparison of genetically resistant and susceptible families at 1 dpi, 7 dpi and 20 dpi was performed using microarrays (Additional file 1). RNA extracted from whole fry homogenates were pooled in four biological replicates (four fry per replicate) per infection status (IPNV-challenged or control) per timepoint per family were used for microarray hybridization. In all cases, the gene expression values of IPNV-challenged samples at each timepoint and genotype were compared to matched controls (such that ‘up-regulation’ refers to a significantly higher gene expression signal in IPNV-challenged fish). Initial analysis showed no indication of systematic bias due to tank effect or day of hybridization. A striking pattern of global gene expression differences between the families was evident, in particular at 7 and 20 days post-challenge, when compared to timepoint and family-matched controls (Fig. 4). The susceptible families showed substantially higher numbers of both up-regulated and down-regulated transcripts than resistant families, and an abundance of transcripts with notably large up-regulation which was not observed in resistant families (Fig. 4). At 1 dpi, the global profile of transcriptional response was similar in terms of magnitude and number of differentially-expressed transcripts, albeit with little overlap in terms of specific differentially expressed transcripts.

**Fig. 5:**
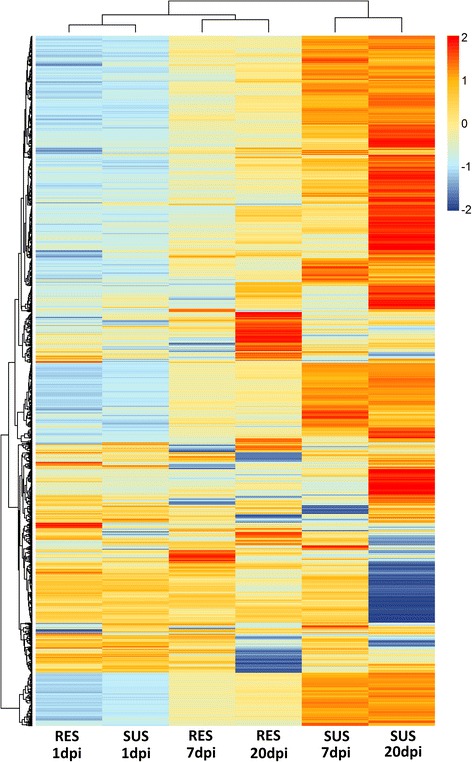
Heatmap of differentially expressed genes in resistant and susceptible families. Heatmap showing the expression of all the differentially expressed genes with log2 fold change > 1 in any of the six comparisons and the clustering of the susceptible and resistant samples at 1, 7 and 20 days post infection

Clustering of gene expression profiles for each timepoint and genotype demonstrated that the susceptible 7 and 20 dpi samples clustered separately from the other genotype x timepoint combinations (Fig. 5). The abundance of highly up-regulated genes is evident from the bias towards red and orange colours for susceptible fish at 7 and 20 dpi which is not observed for other conditions (Fig. 5), consistent with the volcano plots (Fig. 4). The gene expression profile of resistant fish at 7 and 20 dpi fish remained more similar to 1 dpi samples (both resistant and susceptible), which is consistent with a more moderate immune response. Despite this, some resistant-specific sets of differentially expressed genes were observed, which revealed that the differences in the genetic immune response between resistant and susceptible fish are not only quantitative, but also qualitative. The identified set of resistance-specific genes may provide candidates allowing for improved understanding of the functional differences between IPNV-resistant and IPNV-susceptible fish, and of how transcriptomic response determines the outcome of an infection.

### Enrichment analysis

**Fig. 6:**
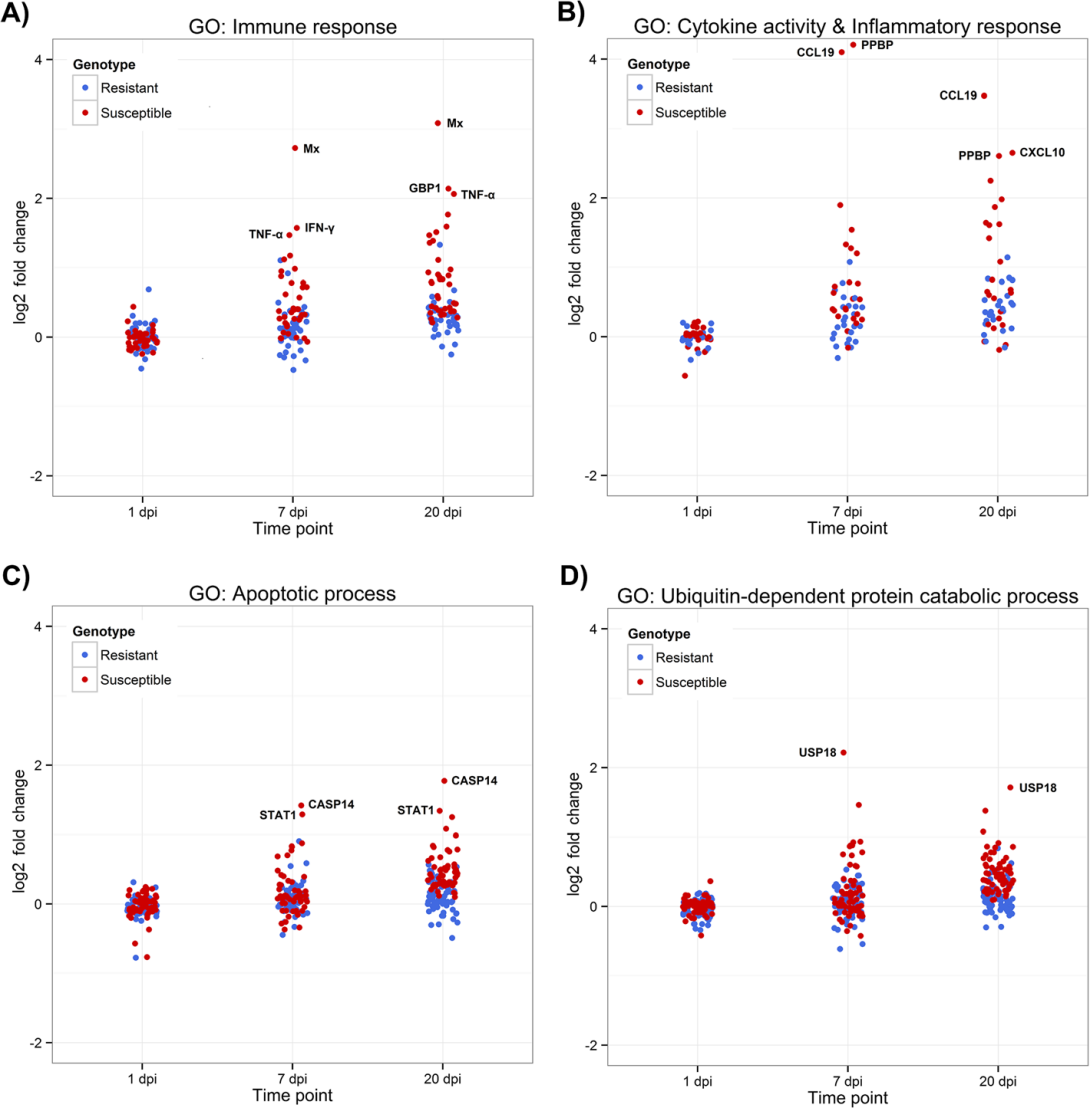
Scatterplots of gene expression for selected enriched GO terms. Scatterplots showing the log2 fold change values of genes differentially expressed at any of the six comparisons and annotated with the GO terms: **a** “immune response”, **b** “cytokine activation” and “inflammatory response”, **c** “apoptotic process”, and **d** “ubiquitin-dependent protein catabolic process”

To examine the observed expression patterns in more detail, GO enrichment analyses were conducted for significantly up- and down-regulated transcripts for each comparison (Additional file 2). Among the GO terms enriched in the up-regulated transcripts, the term “immune response” was (unsurprisingly) clearly enriched in susceptible fish both at 7 dpi and 20 dpi (76 and 95 genes, respectively), while in resistant fish it was only enriched at 20 dpi and with a much lower number of genes (32) (Fig. 6). This pattern is consistent with a heightened immune response in susceptible fish. Further, the enrichment analysis also highlighted several other up-regulated immune-related GO terms which pointed towards a large number of cytokines and other genes involved in inflammation and apoptosis being differentially expressed in susceptible fish but not in those from resistant families (Fig. 6). Similarly, “ubiquitin-dependent degradation” shows a similar pattern and therefore may also play a role in immune defence against IPNV or its dysregulation (Fig. 6). Among the down-regulated genes, many enriched GO terms both in resistant and susceptible families were related to metabolism (e.g., “lipid biosynthetic process”, “tRNA metabolic process” or “tetrapyrrole metabolic process”), which may be related to lower energy availability as an effect of anorexia, one of the symptoms of IPN. A larger number of metabolism GO terms (“glycoprotein metabolic process”, “vitamin metabolic process” or “energy reserve metabolic process) were enriched for differential expression in susceptible individuals, which is consistent with a more severe viraemia. In addition, many terms related to muscle activity were observed only in susceptible individuals at 20 dpi (e.g., “myofibril”, “regulation of muscle contraction” or “muscle myosin complex”), which may explain the abnormal swimming patterns seen in affected fish, another symptom of the disease.

A supplemental enrichment analysis was performed using the KEGG pathway database (Additional file 3). This yielded findings that were consistent with those of the GO enrichment analysis, with several generic viral pathways (e.g., “Measles”, “Influenza A” or “Epstein-Barr virus infection”) and the immune pathway “Toll-like receptor signaling pathway” up-regulated at 7 and 20 dpi in both susceptible and resistant fish, albeit always more intensely in the former. In addition, several up-regulated pathways relating to immune response were observed to be enriched at 7 and 20 dpi in susceptible samples but only at 20 dpi in resistant samples (i.e., “TNF signalling pathway, “cytokine-cytokine receptor interaction” or “chemokine signalling pathway”). Among these were the “RIG-I like receptor signaling" pathway, responsible for detecting viral particles and activating the interferon response; and the “Jak-STAT signaling" pathway, responsible for activating interferon stimulated genes (ISGs). This clearly points towards involvement of interferon response pathways in both susceptible and resistant families, albeit to a greater extent in susceptible fish. In contrast, the coagulation and complement pathway was found to be consistently down-regulated only in susceptible families later in the course of infection (at 20 dpi; Additional file 4). The down-regulation of this pathway may be related to successful deployment of viral host-immunity evasion mechanisms. The fact that the specific and widespread down-regulation of this pathway is unique to susceptible fish at 20 dpi may warrant further investigation.

### Interferons and resistance-associated genes

**Fig. 7:**
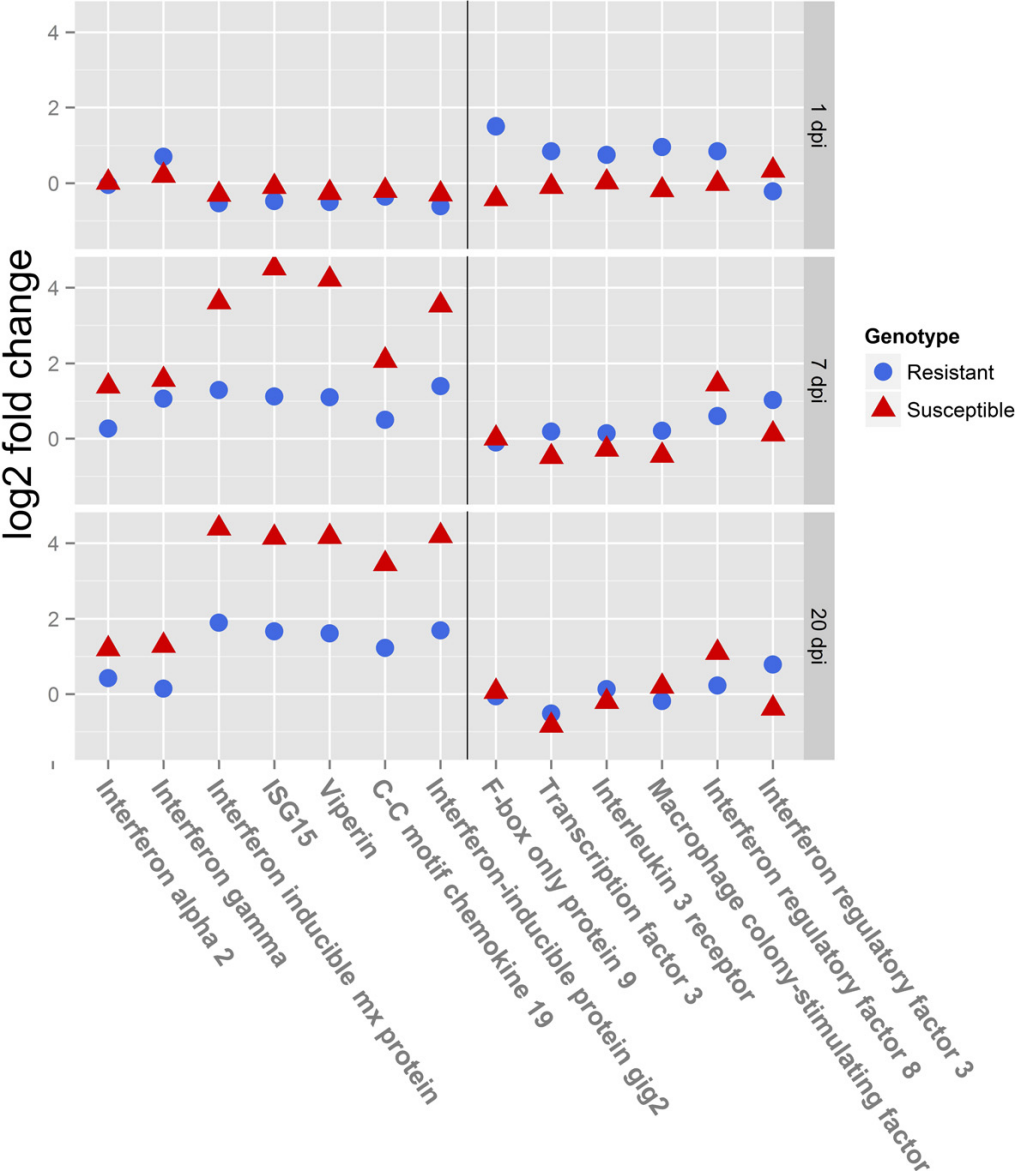
Expression profiles for selected key interferon-related and resistance-specific genes. Log2 fold change values for selected genes in susceptible and resistant families at 1, 7 and 20 days post infection. Genes of interest were selected based on their relationship with the interferon pathway (left of figure) or due to their specific up-regulation in resistant samples (right of figure)

The interferon response pathway is considered the primary antiviral defence system both in fish and in other vertebrates [19] and in particular has been shown to be paramount in host response to IPNV [12–15]. Therefore, the expression patterns of key interferons and ISGs (Fig. 7) were specifically examined. In susceptible fish, the up-regulation of both interferon alpha (IFN-α) and interferon gamma (IFN-γ) was clear at 7 dpi and remained high at 20 dpi. Conversely, resistant fish showed up-regulation of IFN-γ at 1 and 7 dpi, but not at 20 dpi, while IFN-α remained at basal levels throughout the infection. As expected, some of the most important ISGs, specifically interferon inducible Mx protein (Mx), ISG15 ubiquitin-like modifier (ISG15), viperin (vip-2), C-C motif chemokine 19 (CCL19) and interferon-inducible protein gig2 (gig2), were also clearly up-regulated in susceptible samples, with log_2_ fold changes of ~4× both at 7 and 20 dpi. Moderate up-regulation was also observed in the resistant fish at the same timepoints but with log_2_ fold changes ~1–2×. Reyes Lopez et al. [18] also observed a more moderate interferon alpha response in head kidney of resistant fish but, in contrast to the current study, this was only observed at 1 dpi and not by 5 dpi.

To resolve additional details of the basis for the differentiated immune response between phenotypes, the expression profile of some individual transcripts, up-regulated in resistant but not in susceptible samples were specifically examined (Fig. 7). Several of these transcripts have obvious immune functions, like interferon regulatory factors 3 and 8 (IRF3 and IRF8), interleukin 3 receptor (IL3r) or macrophage colony-stimulating factor (M-CSF); while other transcripts, although less well-known, are also connected to the immune system: F-box only protein 9 (FBXO9) and Transcription factor 3 or Transcription factor E2-alpha (TCF3). All these genes showed higher expression in resistant fish at 1 dpi except IRF3, which had higher expression at both 7 and 20 dpi. These genes are functionally connected to myeloid cell lineages and macrophages, which are important components of the cellular immune response.

## Discussion

Resistant and susceptible Atlantic salmon families were identified in order to allow experimental study of the transcriptomic aetiology of heritable resistance to IPN. The existence of a major genetic component to Atlantic salmon resistance to IPN has been extensively documented previously [3–11]. The functional basis of genetic resistance to IPN in salmon has been less studied but is equally important, since it can shed light on the biological mechanisms underlying the genetic resistance and susceptibility. Knowledge of these mechanisms can help understand viral infection processes more generally and improve opportunities for minimizing the potential for impact of viral epizootics in fish.

### Viral load and host genotype

The resistant families in this experiment displayed virtually no losses and may thus be considered completely resistant to mortality as a consequence of IPN. Given that the genetic resistance is almost monogenic [6, 10], the lack of mortality could reflect a matching absence of viral infection following challenge, for example due to the failure of the virus to attach, internalize or replicate in host cells. Indeed, Moen et al. [11] suggest that failure to enter host cells is the primary mechanism underlying the major QTL controlling resistance. However, in the current study, the estimated IPN virus copy number in IPN-resistant salmon strongly imply successful viral replication within host cells. While the underlying QTL genotype of the parents of the resistant families were not known, they were postulated to be homozygous for the resistance allele (‘RR’) [8], and virology results from confirmed ‘RR’ homozygous fish within QTL-segregating families show similar estimated IPNV copy number profiles (unpublished data). A possible reason for the discrepancy between studies is that Moen et al. [11] only described data from sampling the livers of resistant fish at 34 days post-infection, by which stage it is conceivable that the resistant fish had cleared the infection, or the virus may be replicating in other tissues. Reyes-Lopez et al. [18] also reported that both resistant and susceptible fry were infected with IPNV at 1 and 5 dpi, but with higher titres observed in susceptible fish, consistent with the current study. Further, in the current study and in previous transcriptional comparisons between fry showing different IPN resistance phenotypes [17, 18], stimulation of the immune system in response to the virus was reported for both susceptible and resistant samples. Therefore, it is likely that a component of genetic resistance to IPNV is due to differential response of the fish to the virus once they are infected. Indeed, this differential immune response may be connected to host genetic variation impacting on virus attachment, internalization or replication whereby delay in these processes in resistant fish gives the immune system an opportunity to successfully respond to the infection.

### Immune response in susceptible and resistant fish

The observed immune response to IPNV is generally larger and more intense in susceptible fish at 7 and 20 dpi, involving almost every major component of the innate immune system. Resistant fish also showed an immune response to IPNV at both 7 and 20 dpi, but lower in both number of differentially expressed genes and in their intensity of expression. These results are, in part, consistent with those observed by Reyes-López et al. [18], where a high inflammatory response was observed in susceptible but not in resistant fish. However, Reyes-López et al. [18] reported that the initial response at 1 dpi was more intense in susceptible fish but dropped to near basal levels at 5 dpi, with lower values than resistant fish for many immune-related genes. In the current study, the differences between resistant and susceptible genotypes were most marked at 7 days post-infection, and the vast majority of innate immune response genes had higher expression in susceptible fish. In addition, despite using similar timepoints to the current study for sampling, Cofre et al. [17] showed that for eight immune-related genes, including IFN-α and Mx, expression levels were either equal or higher in resistant fish. The differences may be due to the different samples used, with liver or head kidney being examined in previous studies and whole fry examined in the current study. Another potential reason for the differences between studies could be the criteria used to define susceptible and resistant families. The resistant families in the previously published experiments had mortality rates between 15 and 30 % [17, 18], while in our experiment mortality of resistant families was virtually zero, similar to baseline controls. Hence, it may be possible that there are fundamental differences in the response to the IPN virus in families that are fully (current study) or partially (previous studies) resistant. However, it should be noted that mortality level for any given genotype is related to a number of factors including dose response [20], virulence of the viral isolate employed for the challenge and a range of factors relating to previous host history and environment such that even subtle differences in the dynamics of the infection might impact observed mortality.

The inability of susceptible fish to stop the infection seems to produce a disproportionate immune response which includes high representation and expression of inflammatory pathway members, IFN-responsive elements and cytokines leading to eventual apoptosis, with a more controlled immune response being characteristic of resistant fish. In Atlantic salmon challenged with infectious salmon anaemia virus (ISAV), a dramatic up-regulation of transcripts relating to innate immunity was observed in susceptible individuals, which did not provide protection, while resistant individuals were characterized by lower inflammatory response, which allowed fish to survive for longer periods under high viral loads until the activation of pathways associated with adaptive immunity was able to clear the virus [21]. Similarly, bacterial infection by *Piscirickettsia salmonis* in susceptible Atlantic salmon triggered an exacerbated but inefficient immunological response [22]. While inflammation is critical to the efficiency of the innate immune response [23], long-term activation of inflammatory processes can be seriously detrimental to the host [24]. The main site of entry and early replication for IPNV is considered to be the intestinal epithelium [25]. One of the symptoms of IPNV infection in salmon is catarrhal enteritis, and lasting inflammation can produce damage in the intestinal epithelium, as previously suggested for salmon and other species [22, 26–28]. It is possible that a local and effective immune response may be observed in the intestinal epithelium of resistant fish, whereas susceptible fish fail to control the virus at this early stage and it becomes a more systemic infection. Further, intestinal damage and anorexia, causing lower energy availability, may partially explain the down-regulation of metabolic pathways and processes. In a previous study in Atlantic salmon with different levels of flesh n-3 long-chain polyunsaturated fatty acid, a connection between high lipid levels and anti-inflammatory action was suggested [29]. Hence, the down-regulation of lipid metabolism in susceptible fish might contribute to the exacerbation of the immune response. In the current study, the use of whole fry as the sample ensured all tissues were included in the comparison, but obviously has the limitation of precluding the identification of localized and tissue-specific responses and potentially obscuring levels of response.

There was marked and almost universal down-regulation of the complement and coagulation cascade observed in susceptible fish at 20 dpi (but not other timepoints). The complement system plays a major role in viral pathogenesis, including the modulation of both inflammatory and adaptive immune responses [30] and contributes to neutralisation of certain viruses in salmonid fish [31]. There is clear evidence for viral evasion of the host complement system in a wide range of host-pathogen relationships, often including the usurping of host complement regulators by viral homologues, thus highlighting its importance in host response [30]. VHSV infection in rainbow trout, for instance, produces a clear down-regulation of complement genes [32], which might suggest a role for active viral suppression of this pathway. In humans, the complement system is suppressed in individuals persistently infected with hepatitis C and virus-mediated down regulation of the complement system is pertinent to this persistence. Given the fact that susceptible fish which survive up to 20 days have a higher chance of longer-term survival since the most susceptible individuals have already died, and that salmon are known to exhibit an IPNV carrier-state [33], this complement system suppression may be a characteristic of IPNV carriers among susceptible fish.

Transcripts related to ubiquitin-mediated protein degradation were typically up-regulated in susceptible fish but less so in resistant fish. The ubiquitin system plays a critical role in immune defence, having a critical role in antigen presentation [34] and being used as part of the host defence strategy to suppress viral production [35]. In the case of IPNV, manipulation of protein metabolism of the host cell is postulated to be a key viral strategy in evading the interferon-mediated host response [36], and the ubiquitin-proteosome system is likely to contribute to this protein turnover.

### Interferon

The interferon system plays a critical role in both innate and adaptive responses to virus infection. In the current study, a clear up-regulation of genes and pathways associated with the interferon response was observed, both in resistant and susceptible fish, but with this being much more pronounced in the latter. Interferon production and its downstream consequences in the innate immune system are the primary host defence mechanisms against IPNV and other viruses in salmonid fish [13, 31]. ISGs like Mx, ISG15, Vip-2, gig2 or CCL19, all of which were up-regulated in the current study, are among the most well-established IFN-induced genes displaying antiviral properties, and have previously been shown to play a role in host responses to IPNV infection in salmon [12, 18, 37–41]. Mx proteins, previously reported to block IPNV replication in Atlantic salmon cell culture [42], were found to be up-regulated in kidney, liver, spleen and gills of Atlantic salmon challenged with IPNV [43]. In addition, Cofre et al. [17] detected higher expression of Mx in the head kidney of resistant compared to susceptible fish at 1 dpi and of Vig-2 at 1 and 5 dpi, while IFN-α expression was higher in resistant samples at every time point (1, 5 and 21 dpi). Reyes-López et al. [18] detected higher expression of CCL19 in susceptible fish at 1 dpi but this dropped back to basal levels by 5 dpi. Interestingly however, its expression in resistant samples remained moderately up-regulated both at 1 and 5 dpi. This expression pattern was consistent with the expression of IFN-α, which showed a similar profile to CCL19 [18]. Conversely, in the current study, these genes did not show any response at 1 dpi, yet were moderately up-regulated in resistant fish (logFC ~ 2) at 7 and 20 dpi, but highly up-regulated in susceptible (logFC ~4) fish. This suggests that this system alone is unlikely to be able to provide a sufficiently effective response to infection.

A number of IFN-related genes were up-regulated specifically in resistant fish at certain timepoints. For instance, IFN-γ showed higher expression at 1 dpi in resistant fish but not in susceptible fish (Fig. 7). Although the difference was small, it could be biologically relevant, especially considering that IRF8, which was previously shown to respond specifically to IFN-γ in Atlantic salmon [37], was also only up-regulated in resistant fish at 1dpi. It is also worth noting that in resistant fish IFN-α does not seem to be up-regulated at 7 dpi and scarcely at 20 dpi, unlike the case for susceptible fish. Although IFN-γ is a much weaker inducer of ISGs than IFN-α [44], interferon-independent activation of ISGs has been reported in mammals involving IRF3 [45], [46], and this gene, which is only up-regulated in resistant fish at 7 and 20 dpi, has been shown to activate the expression of ISGs in fish [47]. Therefore, perhaps a different IFN-activation route during the early response to IPN infection leads to altered downstream responses and disease outcome. Furthermore, in mammals, INF- γ, also stimulates the host defence by enhancing the function of the proteasome and antigen presentation [48]. Hence it is possible that early up-regulation of IFN-γ in resistant fish may contribute to virus control through an increase in effectiveness of the host ubiquitin-proteosome function and/or by improving the resistance of this pathway to manipulation by the virus. However, while IFN-γ showed potent antiviral activity against salmonid alphavirus 3 (SAV3) in vitro, its antiviral activity against IPNV was found to be lower than that of IFN-α [37]; hence the importance of the two types of interferons in the immune response to IPNV remains unclear.

### Macrophages may be important in defining IPN resistance

IFN-γ is also instrumental in promoting T helper cell response and activating macrophages [49], which show the highest basal expression of the IFN-γ receptor of all cell-types in rainbow trout and zebrafish [50]. Rainbow trout macrophages treated with IFN-γ exhibit an increase in Major Histocompatibility Complex I and II expression, suggesting an important role in enhancing antigen presentation [51–54]. Macrophages show a variety of immunity-related functions, ranging from production of pro-inflammatory or anti-inflammatory cytokines, to phagocytosis and degradation of pathogens and may also be involved in stimulation of the adaptive immune system [55, 56]. IPNV is known to replicate in macrophages of Atlantic salmon [57] and infection is known to stimulate macrophage interferon production [58]. Hence, macrophages may play key roles in the outcome of IPNV infection and this hypothesis is supported by observation of up-regulation of several genes related to macrophage function specifically in resistant families. Macrophage colony stimulating factor (M-CSF) was up-regulated in the resistant but not susceptible families at 1 dpi, and is the principal regulator of survival, proliferation, and differentiation of macrophages and their precursors [59–62]. TCF3 is known to modulate macrophage pro-inflammatory cytokine activity [63], while IL3r and IRF8 are involved in myeloid cell differentiation [64] and maturation [65] respectively. Also FBXO9, which was upregulated in resistant fish at 1 dpi, is a substrate recognition component of a (SKP1-CUL1-Fbox) E3 ubiquitin-protein ligase component which mediates ubiquitination and subsequent proteosomal degradation, blocked the production the pro-inflammatory cytokine Il-6 in mouse macrophages [66].

Macrophages can be activated through a number of different pathways. While the classical M1 pathway leads to a typical inflammatory phenotype, the alternative activation pathway M2 is involved in tissue repair [67]. M2 macrophage activation was shown to be enhanced by the M-CSF [68] which was upregulated in resistant fish in the current study, and IRF3 (up-regulated in resistant fish at 7 and 20 dpi) is known to be diminished in M1-like macrophages and enabled in M2-like macrophages [69]. In contrast, IRF8, up-regulated in susceptible fish at the same timepoints, is characteristic of an M1-like macrophage response [69]. Therefore, M2-type macrophages, and their balance with M1 macrophages, might be involved in the successful immune response of IPNV-resistant Atlantic salmon. Atlantic salmon macrophage activity has been previously reported to increase after ISAV and salmon pancreas disease (SPD) viral infections [70, 71], while a monocyte-macrophage specific gene expression was higher in *Piscirickettsia salmonis* resistant fish than in susceptible fish [22]. The M2 "repair" designation broadly refers to macrophages that function in constructive processes like wound healing and tissue repair, and those that turn off damaging immune system activation by producing anti-inflammatory cytokines. Teleost macrophages were shown to down-regulate inflammatory responses following exposure to apoptotic cells [72]. It is therefore conceivable that the M2 macrophage response may result in better tuning of the immune response in resistant samples, keeping inflammation and apoptosis at appropriate levels and perhaps limiting virus spread by inhibiting their escape from cells. Although the rapid innate immune system may generally be more effective in protecting against RNA viruses [17], it is possible that extending survival of fish until the adaptive immune system can clear the IPN virus, is more important in the present case than a stronger initial innate reaction that could be detrimental to the host. In fact, a lower inflammatory response, combined with an adaptive T-cell response, was suggested to be responsible for survival and ISAV clearance in a challenge test in Atlantic salmon [21].

## Conclusions

IPN resistant and susceptible families were challenged with IPNV to study the differences in their gene expression profiles. While only the susceptible families suffered appreciable mortality, both phenotypes showed significant viral load; hence resistance is apparently not entirely due to the inability of IPN to infect the fish, which is confirmed by the observed immune response in the resistant families. The susceptible fish are characterized by a much larger, yet ineffective, immune response, which involved the production of interferons and other cytokines, and provoked exacerbated inflammation and apoptosis. Resistant fish demonstrated a more moderate response and their gene expression profile pointed to a role of the M2-macrophage system in modulating the inflammatory response, which may contribute to their survival, and partially explain the marked differences between the immune responses of susceptible and resistant families.

## Methods

### Families and IPNV challenge experiment

In order to compare the transcriptomic responses of resistant and susceptible fry families to IPNV challenge, the phenotypes of the families were first defined according to a challenge experiment performed on twenty full sibling families showing diverse IPN resistance breeding values, as calculated using seawater ‘field trial’ data, from the Scottish breeding nucleus of Landcatch Natural Selection Ltd. The details of this first genetics experiment, including the rearing conditions of the fish and the method of IPNV preparation, are given in [8]. Briefly, IPNV isolate V0512-1 [serotype A2 (Sp)] was prepared and harvested using low passage number (P2) in RTG-2 cells. Three replicates of ~ 100 fry from each of the twenty families were transferred to separate 15 L aerated challenge tanks (60 tanks in total) approximately 60 days post hatching. A consistent immersion IPNV challenge (challenge dose ~5.0 x 10^5^ TCID 50 mL^−1^) was applied to two of the three replicates from each family, with the other tank from each family sham-challenged (two challenge and one control tank). The IPNV infection in each tank was then allowed to progress without intervention until there were fewer than three mortalities per day (summed across all of the tanks) for three consecutive days [8].

For the gene expression studies, four families with highest (J & N), and lowest (Q & T) mortality were chosen. The rearing conditions of the fish and method of virus preparation are given in Houston et al. 2010 [8]. Twelve replicates of 50 fry (~95 days post-hatching, mean weight 0.6 g) from each family were transferred from the 15 l holding tanks to 5 l challenge tanks (48 tanks total) maintained at ~ 10 °C. Six of these replicate tanks per family were IPNV-challenged and the remaining six tanks were mock-challenged. The challenge protocol, conditions and monitoring procedure for this experiment were as previously described [8]. However, three timepoints were chosen for termination and sampling of all surviving fry from IPNV challenged and control tanks for subsequent RNA and DNA extraction. These timepoints were informed from the previous challenge experiment and were chosen at 1 day post-challenge (early timepoint), 7 days post-challenge (around the time of the first mortalities) and 20 days post-challenge (around peak daily mortality level). At each of the three timepoints, two IPNV-infected and two control tanks for each family were terminated, with all surviving fish euthanized and then snap frozen in liquid nitrogen and stored at −80 °C until further use. Fish were euthanised using a non-schedule 1 method under a procedure specifically listed on the appropriate Home Office (UK) license and all experiments were performed under approval of Cefas ethical review committee and complied with the Animals Scientific Procedures Act. Mortalities that occurred prior to tank termination were removed and frozen for future IPNV testing.

### IPNV testing

Fry mortalities and survivors from the challenged tanks and control tanks were tested for the presence of IPNV using different methods. Fry were weighed, homogenised using sterile pestle, mortar and sand then diluted 1:10 in cellculture medium. The homogenate was centrifuged at 2500 × g for 15 min. at 4 °C then the supernatant removed and filtered through 0.45 μm filter (Whatman) before inoculation onto 24 h old confluent monolayers of CHSE-214 cells in 96-well cell culture trays for titration according to [73]. Culture trays were incubated at 15 °C and titres read after 7 days. Wells showing positive cytopathic effect (CPE) for each sample were further tested by ELISA (Test-Line) to confirm the presence of IPNV. Subsequently, for the determination of viral load in the samples used for the microarray experiment, an RT-QPCR assay applied in an accredited commercial laboratory (Integrin Advanced Biosystems, UK) was used.

### Microarray platform

Microarray interrogations were performed using a custom-designed, oligonucleotide microarray platform (Agilent) with 44 K probes per slide (Salar_2; Agilent Design ID:025520). This platform has been described in detail elsewhere [74] and used in a number of subsequent studies [29, 75–80], where the expected correlation between microarray fluorescence values and real-time PCR expression has been established. The design is lodged with ArrayExpress (http://www.ebi.ac.uk/arrayexpress) under accession number A-MEXP-2065. Dual-label hybridisations were undertaken, with each experimental sample (Cy3 labelled) being competitively hybridised against a pooled reference control (Cy5 labelled) comprising equimolar amounts from each experimental RNA sample. The interrogations comprised 144 separate hybridisations; 2 genotypes (susceptible, resistant) × 2 families for each genotype × 2 challenge states (control, challenged) × 3 timepoints (1, 7, 20 dpi) × 4 biological replicates for resistant (2 from each of two tanks) and 8 biological replicates for susceptible (4 from each of two tanks). All microarray data has been deposited under accession E-MTAB-4275 in ArrayExpress. A biological replicate comprised four individual fry (see below). A preliminary analysis suggested evidence for a segregating QTL in the susceptible families and therefore twice as many offspring were screened. It was later established that the evidence for a segregating QTL in these families was inconclusive and therefore comparisons were made at the family level only. A direct comparison of QTL genotypes within families has been conducted using a combination of microarray and RNA-seq in other families, which will be reported separately. The analyses took the unbalanced design into account.

### RNA extraction and purification

From the −80 °C stored samples, 12 survivors from each tank were randomly selected for microarray analysis. Whole fry (*n* = 576) were homogenised in 8 volumes of TRI Reagent (Sigma–Aldrich®, St. Louis, U.S.A.) using a Polytron mechanical homogeniser (Kinematica PT 1300 D, Lucerne, Switzerland) and the RNA extracted following the manufacturer’s instructions. RNA quantity and quality were assessed by spectrophotometry (NanoDrop ND-1000, Thermo Scientific, Wilmington, U.S.A.) and agarose gel electrophoresis respectively. Equal amounts of RNA from four individuals, sourced from the same tank were pooled to form each biological replicate. The RNA from each pool (*n* = 144) was column-purified (RNeasy Mini Kit, Qiagen, Crawley, UK), and then re-quantified and quality assessed as described above.

### RNA amplification and labelling

Each pooled RNA sample was amplified (TargetAmp^TM^ 1-Round Aminoallyl-aRNA Amplification Kit, Epicentre Technologies Corporation, Madison, Wisconsin, USA) according to the manufacturer’s instructions. Following QC (Nanodrop quantification and agarose gel electrophoresis) a reference (pool) sample was created by combining an equal amount of aRNA from each of the 144 reactions. Each aRNA sample was indirectly labelled (Cy3) and purified, while a similar (Cy5) labeling was undertaken for aliquots of the pooled reference sample. Briefly, Cy dye suspensions (Cy3 and Cy5) were prepared by adding 44 μL high purity dimethyl sulphoxide (Stratagene, Hogehilweg, The Netherlands) per tube of Cy dye (PA23001 or PA25001; GE HealthCare, Little Chalfont, Bucks, UK). Each aRNA (2.5 μg) was denatured at 70 °C for 2 min in 10.5 μL water and then 3 μL 0.5 M NaHCO_3_ pH8.5 and 1.5 μL Cy dye (Cy3 or Cy5) was added and gently mixed. The suspension was incubated for 1 h at 25 °C in the dark and the excess label was removed by spin-column purification (Qiagen GE Healthcare). Dye incorporation and purity were assessed via spectrophotometer (NanoDrop) and, following agarose gel (1 %) electrophoresis, aliquots of the labelled aRNA were also visualised on a fluorescent scanner (Typhoon Trio, GE Healthcare).

### Microarray hybridization and quality filtering

Hybridisation was performed over 6 days (24 hybridisations per day) using proprietary apparatus and solutions (Agilent Technologies) as per manufacturer’s instructions. For each reaction, 825 ng Cy5 labelled reference pool and 825 ng Cy3 labelled individual sample were combined in 35 μL nuclease free water and then 20 μL fragmentation master mix added (comprising 11 μL of 10X blocking agent, 2 μL 25x fragmentation buffer and 7 μL nuclease free water). The reactions were then incubated at 60 °C in the dark for 30 mins, chilled on ice, and mixed with 55 μL 2x GEx Hybridisation buffer (pre heated to 37 °C). Following centrifugation (18000 × g for 1 min) the samples were kept on ice until loaded (103 μL) in a semi randomised order onto the microarray slides. Similar numbers of samples from the different states, treatments and families were distributed across slides (*n* = 6) each day. Hybridisation was carried out in a rotating oven (Agilent Technologies) at 65 °C, 10 rpm for 17 h.

Following hybridisation, slides were subject to a number of washing steps performed in Easy-Dip^TM^ slide staining containers (Canemco Inc., Quebec, Canada). First, each microarray and backing gasket were disassembled in Agilent Wash Buffer 1 and microarray slides were transferred to an Easy Dip Rack submerged in Wash Buffer 1. Following 1 min. incubation at room temperature (c. 20 °C) and 150 rpm (Stuart Orbital Incubator), slides were briefly dipped into Wash Buffer 1 pre-heated to 31 °C, then placed into Wash Buffer 2 (31 °C) for 1 min at 150 rpm. Finally, the slides were transferred to acetonitrile for 10 s. and then Agilent Stabilization and Drying Solution for 30 s. The slides were then air dried in the dark and scanned within two hours.

Scanning was carried out at 5 μm resolution on an Axon GenePix Pro scanner (Axon Instruments Inc.) at 40 % laser power. The “auto PMT” function was enabled to adjust PMT for each channel such that less than 0.1 % of features were saturated and so that the mean intensity ratio of Cy3:Cy5 signal was close to one. Agilent Feature Extraction Software (v 9.5) was used to identify features and extract background subtracted raw intensity values that were then transferred to GeneSpring GX (v12) software where the quality filtering and normalisation steps took place. Intensity values ≤ 1 were adjusted to 1 and a Lowess normalisation undertaken. Stringent quality filtering ensured that features that represented technical controls, saturated probes, probe population outliers or probes which were not significantly different from the background were removed. This left 33,688 of the original 43,466 probes available for downstream analysis and a two-way unbalanced ANOVA was performed in the Genespring software (Agilent, CA, USA) to assess differential expression. The data will be submitted to arrayexpress prior to publication.

### Enrichment analyses

Gene ontology (GO) enrichment analysis was performed using BLAST2GO [81] and KEGG pathway enrichment using KOBAS 2.0 [82], using the total microarray probes as background. Enrichment probability values for BLAST2GO and KOBAS analyses were adjusted for multiple testing (FDR-corrected P-values < 0.05) to consider significantly over-represented GO-terms and KEGG pathways within each list of differentially expressed genes. Heatmap, scatterplots and gene fold change graphs were created using R v.3.0.1 [83] and the R packages NMF [84] and ggplot2 [85].

### Availability of data and material

The datasets supporting the conclusions of this article are available in the ArrayExpress repository MTAB-4274 (https://www.ebi.ac.uk/arrayexpress/experiments/E-MTAB-4275/).

## Additional files

Additional file 1: Differentially expressed genes in all comparisons. List of differentially expressed genes in all comparisons. Probe name, p-value, fold change,up- or down-regulation, annotation, transcript sequence and probe sequence are shown. (XLSX 7694 kb) Additional file 2: Enriched GO terms in all comparisons. List of enriched Gene Ontology (GO) terms in all comparisons. GO terms IDs, GO term description, GO category (P: biological process; M: molecular function; C: cellular component), false discovery rate corrected p-value, number of genes with the corresponding GO term among the differentially expressed genes and their microarray IDs, and number of genes with the corresponding GO term in the whole microarray and their microarray IDs are shown. (XLSX 1044 kb) Additional file 3: Enriched KEGG pathways in all comparisons. List of enriched Kyoto Encyclopedia of Genes and Genomes (KEGG) pathways in all comparisons. KEGG pathway description, KEGG pathway ID, number of differentially expressed genes assigned to the corresponding KEGG pathway and their microarray IDs, number of genes assigned to the corresponding KEGG pathway in the whole microarray, false discovery rate corrected p-value and hyperlink to a visual representation of the KEGG pathway with the differentially expressed genes outlined are shown. (XLSX 92 kb) Additional file 4: Scatterplot of gene expression for the complement and coagulation cascade KEGG pathway. Scatterplot showing the log2 fold change values of genes differentially expressed at any of the six comparisons and annotated to the KEGG pathway “complement and coagulation cascades”. (PNG 243 kb)

## Abbreviations

BBSRC: Biotechnology and Biological Sciences Research Council
CCL19: C-C motif chemokine 19
CDH1: cadherin-1 gene
CPE: cytopathic effect
dpi: days post infection
FBXO9: F-box only protein 9
gig2: interferon-inducible protein gig2
GO: gene ontology
IFN-α: interferon alpha
IFN-γ: interferon gamma
IL3r: interleukin 3 receptor
IPN: infectious pancreatic necrosis
IPNV: infectious pancreatic necrosis virus
IRF: interferon regulatory factor
ISAV: infectious salmon anaemia virus
ISG: interferon stimulated gene
ISG15: ISG15 ubiquitin-like modifier
MASTS: The Marine Alliance for Science and Technology for Scotland
M-CSF: macrophage colony-stimulating factor
Mx: interferon inducible Mx protein
QTL: quantitative trait locus
SAV3: salmonid alphavirus
SPD: salmon pancreas disease
TCF3: transcription factor 3
vip-2: viperin
