# Supplementary figures and images for: Gene expression comparison of resistant and susceptible Atlantic salmon fry challenged with Infectious Pancreatic Necrosis virus reveals a marked contrast in immune response
Source: BMC Genomics. 2016 Apr 11;17:279. doi: 10.1186/s12864-016-2600-y (PMC4827185; doi:10.1186/s12864-016-2600-y)

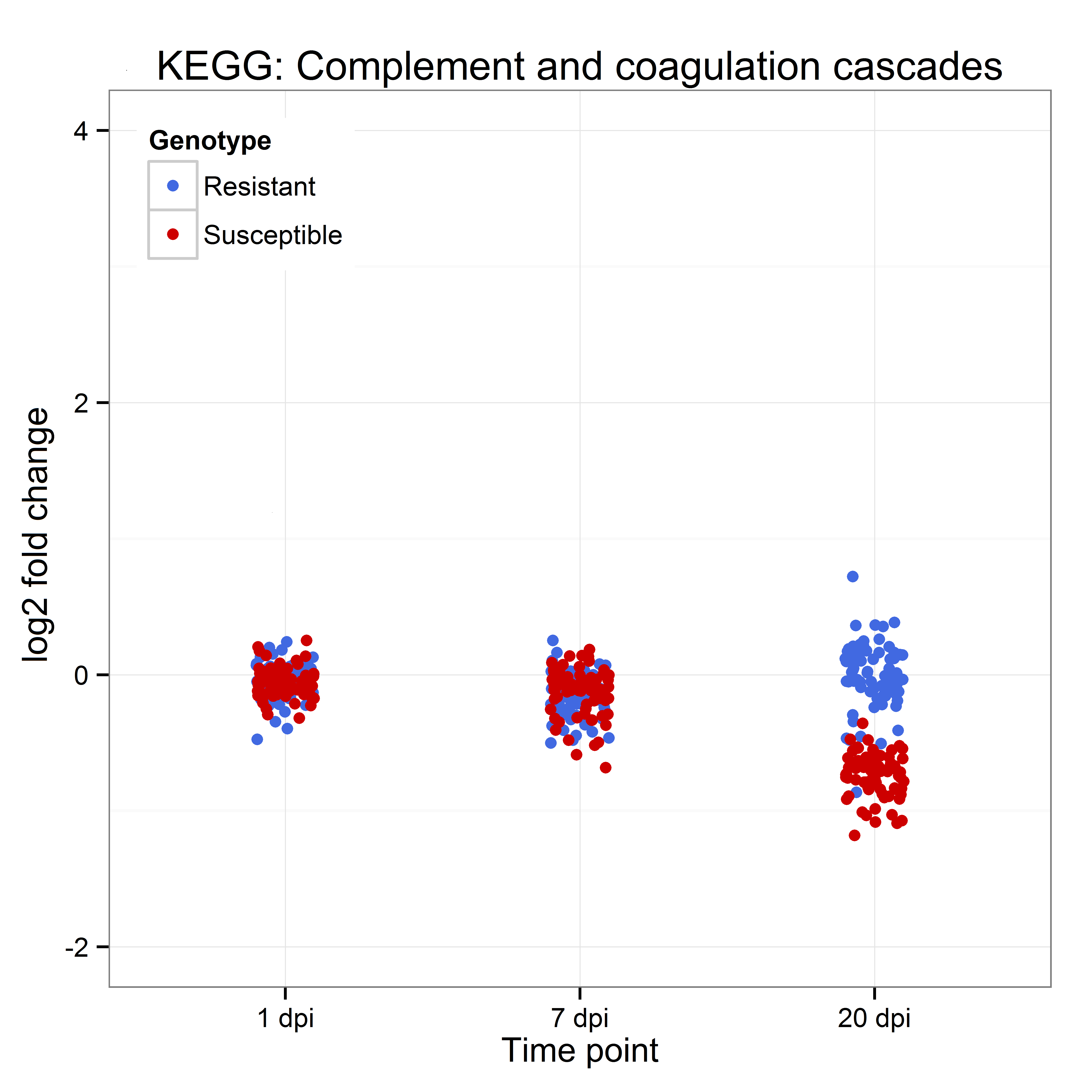

Supplement: Additional file 4: — Scatterplot of gene expression for the complement and coagulation cascade KEGG pathway. Scatterplot showing the log2 fold change values of genes differentially expressed at any of the six comparisons and annotated to the KEGG pathway “complement and coagulation cascades”. (PNG 243 kb) [file 12864_2016_2600_MOESM4_ESM.png]
